# Functional interaction between co-expressed MAGE-A proteins

**DOI:** 10.1371/journal.pone.0178370

**Published:** 2017-05-24

**Authors:** Julieta E. Laiseca, María F. Ladelfa, Javier Cotignola, Leticia Y. Peche, Franco A. Pascucci, Bryan A. Castaño, Mario D. Galigniana, Claudio Schneider, Martin Monte

**Affiliations:** 1 Lab. Oncología Molecular, Departamento de Química Biológica and IQUIBICEN-UBA/CONICET, Facultad de Ciencias Exactas y Naturales, Universidad de Buenos Aires, Buenos Aires, Argentina; 2 Lab. Inflamación y Cáncer, Departamento de Química Biológica and IQUIBICEN-UBA/CONICET, Facultad de Ciencias Exactas y Naturales, Universidad de Buenos Aires, Buenos Aires, Argentina; 3 Laboratorio Nazionale del Consorzio Interuniversitario per le Biotecnologie, Area Science Park, Trieste , Italy; 4 Lab. Biología Molecular y Celular, Departamento de Química Biológica, Facultad de Ciencias Exactas y Naturales, Universidad de Buenos Aires, Buenos Aires, Argentina; 5 Laboratorio Receptores Nucleares, IBYME-CONICET, Buenos Aires, Argentina; 6 Dipartimento di Scienze e Tecnologie Biomediche, Università di Udine, p.le Kolbe 4, Udine, Italy; University of Naples 2, ITALY

## Abstract

MAGE-A (Melanoma Antigen Genes-A) are tumor-associated proteins with expression in a broad spectrum of human tumors and normal germ cells. MAGE-A gene expression and function are being increasingly investigated to better understand the mechanisms by which MAGE proteins collaborate in tumorigenesis and whether their detection could be useful for disease prognosis purposes. Alterations in epigenetic mechanisms involved in MAGE gene silencing cause their frequent co-expression in tumor cells. Here, we have analyzed the effect of MAGE-A gene co-expression and our results suggest that MageA6 can potentiate the androgen receptor (AR) co-activation function of MageA11. Database search confirmed that MageA11 and MageA6 are co-expressed in human prostate cancer samples. We demonstrate that MageA6 and MageA11 form a protein complex resulting in the stabilization of MageA11 and consequently the enhancement of AR activity. The mechanism involves association of the Mage A6-MHD domain to MageA11, prevention of MageA11 ubiquitinylation on lysines 240 and 245 and decreased proteasome-dependent degradation. We experimentally demonstrate here for the first time that two MAGE-A proteins can act together in a non-redundant way to potentiate a specific oncogenic function. Overall, our results highlight the complexity of the MAGE gene networking in regulating cancer cell behavior.

## Introduction

Proteins of the MAGE-A (Melanoma Antigen GEnes-A) family belong to the Cancer Testis Antigens (CTA) group and are therefore expressed in normal testis and a broad spectrum of human tumors [1]. The first MAGE-A members were discovered as a human melanoma tumor antigens [2], although their expression was later confirmed in almost all human cancers. The molecular signature of the MAGE protein family is MHD (MAGE Homology Domain). This conserved domain covers approximately 200 amino acids, contains two structurally similar regions (winged helix 1 and winged helix 2) and could be involved in protein-protein interactions [3].

Since its discovery, MAGE-A tumor-specific expression was firstly exploited to develop tumor vaccines [4]. Studies on MAGE-A protein function started a decade later. Nowadays, growing body of evidence reported by our and other groups suggest that MAGE-A expression might actively contribute to oncogenesis and refraction to chemotherapy [5]. Consistently, clinical observation indicates a correlation between MAGE-A gene expression and poor prognosis [6,7].

In the last years, different MAGE-A proteins have been shown to regulate key cancer-related pathways by targeting proteins such as SKIP [8], the p53 tumor-suppressor [9–11], Mdm2 [12], PML-IV [13], E2F1 [14] or AMPK [15]. Significant to this work, Elisabeth Wilson’s group has extensively worked on MageA11 functional and biochemical characterization. Part of their findings demonstrate that MageA11 is a co-regulator of the Androgen Receptor (AR) and overexpressed in human prostate cancer samples [16–19]. AR is a testosterone-activated transcription factor and a crucial protein in prostate cancer [20].

MAGE-A gene expression in normal somatic cells is repressed by epigenetic mechanisms. Whereas in untransformed cells MAGE-A genes are silenced, frequent epigenetic reprogramming in tumor cells leads to global DNA hypomethylation and MAGE-A expression [21,22]. Moreover, induction of DNA hypomethylation can drive oncogenesis in mice [23]. Both biochemical and genetic approaches strikingly support the notion that MAGE-A genes are highly sensitive to both histone and DNA methylation status in their promoters [24–26]. Global epigenetic changes in tumor cells could induce the well-documented co-expression of MAGE-A genes in a tumor sample. However, investigation on the effect of MAGE-A protein co-expression has not been reported yet.

Here, as result of our attempt to understand the connotation of MAGE-A protein co-expression in tumor cell behavior, we have unexpectedly found that they can interact to potentiate a specific MAGE-A function. Even when co-expression of MAGE-A proteins could hypothetically cause a variety of anomalies due to the sum of their individual actions on different cancer-related pathways, we describe here for the first time that two MAGE-A members can potentiate a single cancer-associated function by playing specific roles. We observe that MageA6 can stimulate AR through a Mage-A11-dependent way. The mechanism involves protection of MageA11 ubiquitinylation and its stabilization by MageA6 expression.

## Materials and methods

### Cell culture and reagents

HEK293T and LNCaP cell lines were obtained from the ATCC and cultured as recommended. Cycloheximide and MG132 were purchased from Sigma-Aldrich.

### Plasmids, transfections and retroviral infections

HEK293T were transfected with PEI (Polysciences). Plasmids: pcDNA-HA-MageA6 was obtained by cloning MageA6 cDNA into pcDNA-HA vector. GFP-MHD-MageA6 was obtained by subcloning MageA6 cDNA encoding amino acids 110 to 304 into pEGFP-C1.

The following plasmids were previously described: pCMV5-hAR, pcDNA3-flag-ratMR and pSV2Wrec-GR; pCMV5-Flag-MageA11 [17], and pcDNA-HA-MageA2 and pcDNA-HA-ubiquitin [9].

Flag-MageA11^K240A;K245A^ (MageA11-2KA) was obtained from pCMV5-Flag-MageA11 by using the QuickChage II XL Site-Directed Mutagenesis Kit (Agent Technologies). Primer sequences used for mutagenesis were: forward primer 5´-CGAGTCGCGGGGCTGATCACAGCGGCAGAAATG and reverse primer 5´-TTCTGCCGCTGTGATCAGCCCCGCGATTCGATA.

Retroviral plasmid pLPC-MageA6 was obtained by subcloning MageA6 cDNA into pLPC vector (Puromycin resistance). Retroviral infections were performed as described in [13]. For MageA6 silencing we used Mage-A siRNA (h) (sc-35843) and the control siRNA-A (sc-37007) (Santa Cruz Biotech). This reagent does not affect MageA11 expression (data not shown).

### Gene reporter assays

HEK293T cells were seeded in 12 wells plates and transfected with the PSA-Enh-Luc (700ng/well) [27] to test AR (25 ng/well) activity and the pMMTV-Luc (600 ng/well) (mouse mammary tumor virus promoter vector) reporter to test GR (50 ng/well) and MR (50 ng/well) activity. Plasmids encoding MAGE proteins were transfected at 100–200 ng/well. pRL-CMV reporter (70 ng/well) (Promega) was used for normalization.

After 24h post transfection, nuclear receptors were induced with Dexametasone (Dx), Aldosterone (Aldo), or Dihidrotetosterona (DHT) at final concentration of 10^-8^M.

### Antibodies

Western blot analysis was performed according to standard procedures using the following primary antibodies: anti-AR (N-20) (sc-816, Santa Cruz Biotechnology) anti-pan-MageA (6C1, Santa Cruz Biotechnology), anti-actin (13E5, Cell Signal Technology), anti-MageA11 SAB1410769 (Sigma-Aldrich) and anti-β-tubulin (D10, Santa Cruz Biotechnology). For tags we used the following: anti-HA 12CA5 monoclonal antibody (Roche) and anti-FLAG M2 monoclonal antibody (Sigma-Aldrich). Anti-GFP was an affinity-purified polyclonal antibody raised against GST-GFP [28].

### Immunoprecipitation

Cells were harvested in ice-cold lysis buffer containing 50mM Tris-HCl pH 8.0, 150mM NaCl, 1% NP-40, 0.1mM sodium orthovanadate, 2mM DTT, 0.1mM PMSF, 5mM EDTA and Protease Inhibitor Cocktail (Sigma). After 10 min of rocking at 4°C, lysates were clarified by centrifugation and incubated with anti-FLAGM2 antibody and Protein A-Sepharose CL-4B (GE Healthcare Biosciences) for 3h at 4°C. The resin was then washed and bound proteins were eluted in SDS-PAGE sample buffer.

### Quantitative RT-PCR

Total RNA was extracted with RNAzol^R^ RT (MRCgene) and cDNA was transcribed with Im-Prom-II reverse transcription kit (Promega) according to the instructions of the manufacturer. Real-time PCR was performed with SYBR Mix qPCR (ROCHE) and a Stratagene Mx3000 real time PCR machine. Primer sequences were as follows: MageA11 (forward) 5´-GGAGACTCAGTTCCGCAGAG-3´ and (reverse) 5´- TGGGACCACTGTAGTTGTGG-3´; and GAPDH (forward) 5´-ACAGCCTCAAGATCATCAG-3´ and (reverse) 5´-GAGTCCTTCCACGATACC-3´; PSA (forward) 5’-TGAACCAGAGGAGTTCTTGAC-3’ and 5’-CCCAGAATCACCCGAGCAG-3’ (reverse).

### *In vivo* ubiquitinylation assay

HEK293T cells were transfected with plasmids pCMV5-Flag-MageA11 or pCMV5-Flag- MageA11 ^K240A;K245A^ (MageA11-2KA) and pcDNA-HA-Ub in the presence or absence of pcDNA-HA-MageA6. 6 h post transfection cells were washed and 10 μM MG132 was added. After 24 h, immunoprecipitation was performed using anti FLAG M2 antibody, as described above. MageA11 or MageA11^K240A;K245A^ ubiquitinylated forms were detected by western blot using an anti-HA antibody.

### Cycloheximide chase analysis of protein degradation

For cycloheximide chase analysis, HEK293T cells were transfected with pCMV5-Flag-MageA11 (1.5 μg/p35 mm) or pCMV5-Flag-MageA11-2KA (1.5 μg/p35 mm) in the presence or absence of pcDNA-HA-MageA6 (1 μg/p35 mm). In all cases a plasmid encoding GFP (20 ng/p35 mm) was co-transfected as an internal control. After 24 h, cells were incubated with 100 μM of cycloheximide for indicated times.

### Bioinformatics

We browsed the public database cBioPortal for Cancer Genomics [29,30]. This portal collects next generation sequencing data from The Cancer Genome Atlas (TCGA) and the International Cancer Genome Consortium (ICGC). We selected a study including tumor and normal tissue samples [31]. We selected two studies that included gene expression data from RNAseq or gene expression microarray: 1) Prostate Adenocarcinoma (MSKCC, Cancer Cell 2010), and 2) Testicular Germ Cell Cancer (TCGA, Provisional). We used the cBioPortal online interphase to determine MAGEA6 and MAGEA11 expression in prostate tumors (last accessed: sep. 26^th^ 2016). Gene expression levels were calculated as the relative expression of an individual gene to the gene's expression distribution in a reference population. The reference population is all samples that are diploid for the gene in question (by default for mRNA), or normal samples (when specified), or all profiled samples. The returned value indicates the number of standard deviations away from the mean of expression in the reference population (Z-score).

In addition, we plotted and calculated the Spearman correlation coefficient between MAGEA11 expression and other MAGEA genes.

In all cases, protocol approvals and informed consents were obtained by the authors of the original studies.

## Results and discussion

### MageA6 potentiates AR activation in a MageA11-dependent fashion

**Fig 1:**
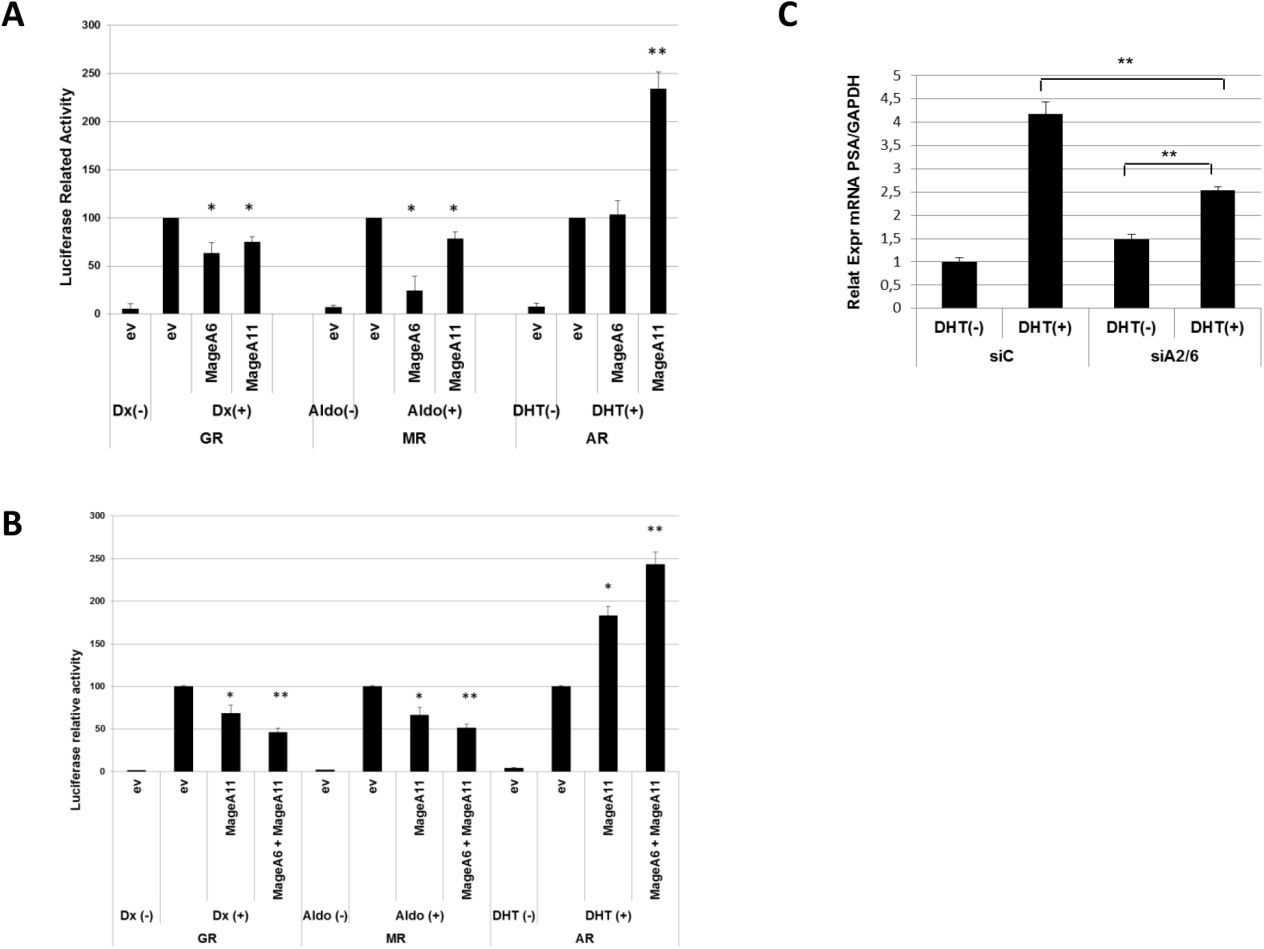
MageA6 enhances MageA11-dependent AR transcriptional activity. (A) Reporter gene assay for GR, MR and AR activity using specific gene-reporter in the presence or absence of MageA6 or MageA11 expression. Cells were treated with dexamethasone (Dx), Aldosterone (Aldo) or dihidrotestosterone (DHT) for 24 h prior to harvesting. The assay was performed in HEK293T cells. ev, empty vector. (B) Similar to A but combining MageA11 and MageA6 expression as indicated. (C) Determination of PSA mRNA levels through RT-qPCR. LNCaP cells were transfected with a siRNA control (siC) or a siRNA to silence MageA6 expression (siA6/2). DHT was added to cells as indicated. PSA mRNA levels were normalized to GAPDH mRNA levels. Error bars indicate mean S.D. Student’s t test was used for statistical analysis. * p < 0.05. ** p < 0.001.

We approached the investigation on MAGE-A specificity in co-expression using two well-characterized readouts such as MageA2 regulation of p53 activity [9] and MageA11 regulation of AR activity [17]. We regularly tested the effect of expression of different MAGE-A genes, either alone or in co-expression, using p53 and AR reporter systems. While the expression of most tested MAGE-A genes affected in some way the activity of p53, we observed an interesting degree of specificity of MAGE-A expression on the regulation of AR activity. Recent unpublished studies form our laboratory evidenced that MageA6 is able to regulate transcription factors of the nuclear receptor (NR) family such as the glucocorticoid receptor (GR) and the mineralocorticoid receptor (MR), but no significant regulation was observed for AR. Therefore, we compared the effect of MageA6 and MageA11 co-expression on the biological response mediated by GR, MR and AR nuclear receptors (NRs). We approached this topic by using specific reporter-gene assay in HEK293T cells, since these cells do not express MAGE genes and do not display significant NRs activity. We observed that MageA6 and MageA11 repressed GR and MR transactivation function. In turn, while MageA6 has no effect on AR function, MageA11 clearly enhanced AR transactivation function as expected (**Fig 1A**). These results indicate that AR behaves differentially when compared to other nuclear receptors upon MAGE-A expression and that MageA6 is not able to enhance AR activity as MageA11 does.

We then tested the potential effect of MageA6 and MageA11 co-expression on all three NRs. The results showed in **Fig 1B** indicate that MageA11 and MageA6 co-expression reinforced their repressive activity on GR and MR, however, when AR activity was analyzed we observed that MageA6 expression collaborates with MageA11 to enhance AR activity Since no direct effects of MageA6 on AR activity were observed, this result suggests that MageA6 could affect AR activity depending on MageA11 expression. Finally, to address whether endogenous MageA6 could regulate AR activation in human prostate cancer cells (LNCaP) expressing MageA11, we silenced MageA6 expression through siRNA-mediated knock-down (KD) and AR function was determined by measuring PSA gene expression levels in DHT treated cells. As shown in Fig 1C, PSA mRNA is decreased in MageA6 KD cells. During our experimental settings, we observed that siA6/2 also diminishes MageA2 levels, but does not affect MageA11 expression (not shown).

Potentially, co-expression of MageA6 and MageA11 could be highly relevant in prostate cancer biology due to their effect on AR. For that reason we next studied MageA6 and MageA11 co-expression in prostate cancer samples.

### Database search for MAGE genes co-expression

**Fig 2:**
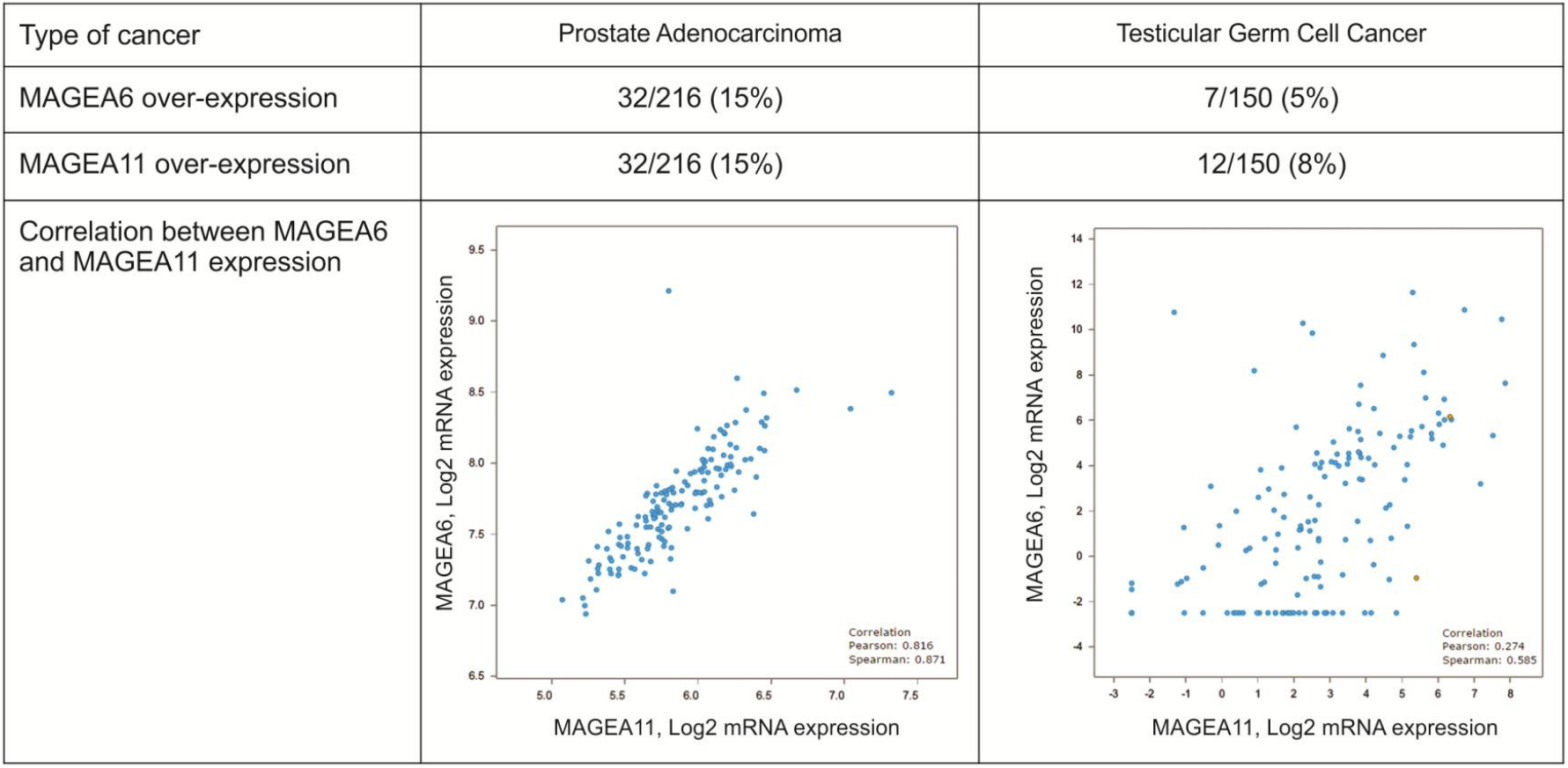
MageA6 and MageA11 co-expression in prostate cancer. Analysis of cBioPortal Cancer Genomics data sets form Prostate Adenocarcinoma and Testicular Germ Cell Cancer (see Material and Methods) as indicated in the two main columns. Gene overexpression was calculated by Z-score, defined as the relative expression of an individual gene to the gene’s expression distribution in a reference population. The indicated percent of over-expression refers to the number of samples over-expressing a given gene over the total of samples. Dot-plot graphics shows the correlation between MageA6 and MageA11 gene expression. Insets indicate Pearson and Spearman correlation scores.

**Fig 3:**
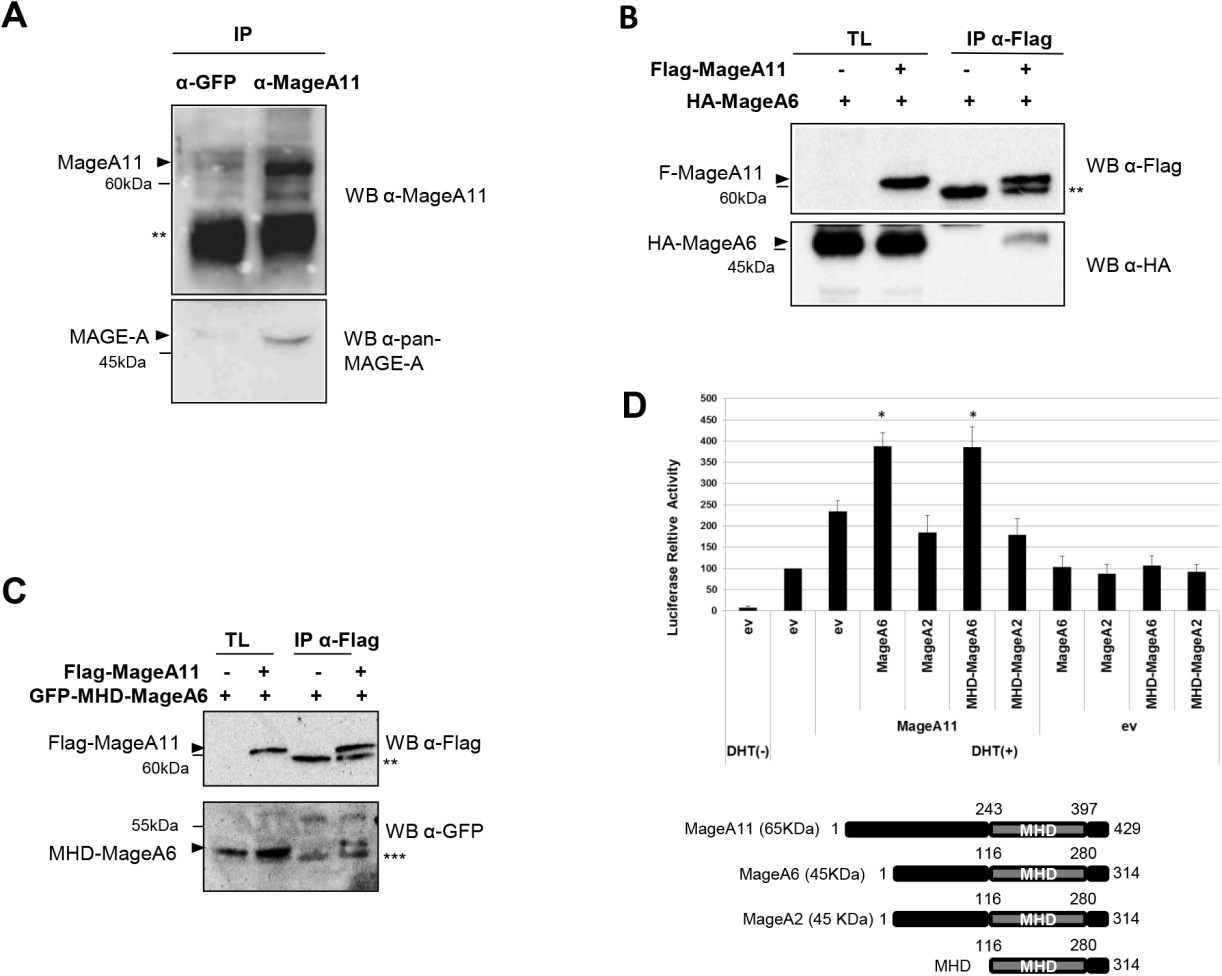
MageA6 associates with MageA11 in cells. (A) Co-immunoprecipitation of MageA11 and Mage-A proteins from LNCaP cells. Specific anti-MageA11 antibody was used to immunoprecipitate MageA11. Western blot was performed using a pan-MAGE-A antibody. Anti-GFP was used as IP control antibody. (B) Co-Immunoprecipitation of Flag-MageA11 and HA-MageA6 form HEK293T cells. Immunoprecipitated protein complexes obtained with the anti-Flag antibody (IP anti-Flag) were assessed by Western blot and probed with the indicated antibodies. TL, total lysates. (C) Similar to A but using Flag-MageA11 and GFP-MHD-MageA6. (D) Upper panel: Reporter gene assay for AR activity in HEK293T cells using the PSA-Luc reporter construct and the plasmid encoding AR. MageA11, MageA6, MageA2 and MHD-MageA6 or MHD-MageA2 were co-transfected as indicated in the figure. Cells were treated with DHT 24 h prior to harvest. ev, empty vector. Bottom panel: A sketch of Mage-A protein domains and their expected MW. Error bars indicate mean S.D. Student’s t test was used for statistical analysis. * p < 0.001; ** Immunoglobulin heavy chain. *** Immunoglobulin light chain. Triangles show the corresponding protein band and dashes mark the MW.

To validate our findings, we analyzed public data of gene expression form the cBioPortal database for human cancer genomics [29,30]. We limited our search to MAGE-A gene co-expression in human prostate cancer due to the relevance of AR activity and in testicular cancer since it is also a hormone-associated male cancer. As shown in **Fig 2,** gene expression data from 216 prostate cancer samples indicates that 15% of them overexpress both genes with high degree of correlation as determined by Spearman’s and Pearson’s rank correlation coefficients. In testicular cancer, MageA6 and MageA11 are overexpressed in 5% and 8% of cases, respectively, with a lower correlation coefficient. Interestingly, when we analyzed the list of MAGE-A genes co-expressed with MageA11 in prostate cancer, we found MageA6 as a top ranked gene together with MageA5 and MageA9 (not shown). MageA5 gene theoretically codifies for a truncated form of a MAGE-A protein that could express only the first N-terminal 124 amino acids. MageA9 protein function has not been characterized yet. Its gene expression has been associated to poor prognosis in different human tumors; nevertheless, these studies do not include prostate cancer data. MageA2 and MageA4 also displayed good correlation with MageA11 expression; however, we observed they have no effect on the MageA11/AR system (Fig 3D and data not shown).

Then, the potential relevance of MageA6 and MageA11 co-expression in prostate cancer prompted us to further study the mechanisms by which MageA6 could enhance MageA11 co-activation on AR.

### MageA6 forms a protein complex with MageA11 through its MHD domain and potentiates AR activity

It has been recently reported that the MHD domain of MageA3 (it shares 98% of amino acid sequence identity with MageA6), but not that of MageA4, forms homodimers in solution [3]. In addition, we and other groups have mapped the MHD domain as a key region for MAGE-A protein-protein interaction. Structural approaches have confirmed this findings [3,32]. Moreover, novel data shows that two conserved and structurally similar regions belonging to the MageA3 MHD domain (winged helix 1 and winged helix 2) can interact each other [3], suggesting that some MAGE-A proteins could homodimerize through their MHD domains.

Based on this data, we hypothesized that in the case of co-expression, two MAGE-A proteins could also interact to form heterodimers in cells. To analyze this hypothesis, we immunoprecipitated (IPed) MageA11 protein from LNCaP cells using an anti-MageA11 antibody. The presence of MageA6 was then assessed by western blot using a pan-MAGE-A antibody. **Fig 3A** shows a band corresponding to MageA6 MW (45KDa) co-immunoprecipitated with MageA11. However, since MageA6 cannot be recognized by specific antibodies, and other Mage-A proteins share similar MW, we confirm the association between MageA6 and MageA11 using transfected tagged proteins. Tagged proteins were co-expressed in HEK293T cells and the immunoprecipitation was carried out using anti-tag antibody. As shown in **Fig 3B**, MageA6 was found associated to MageA11 in a protein complex. Then, we performed a similar IP assay using a construct expressing only the MageA6-MHD and MageA11. To this aim, we replaced the N-terminal region (1–109 amino acids) of MageA6 with the GFP protein. As observed in **Fig 3C**, MageA11 and MageA6 MHD interact in cells, indicating that MageA6 can associate through its MHD domain to MageA11.

To investigate the mechanism by which MageA6 stimulates MageA11 activity, we tested the effect of MageA6-MHD on MageA11-dependent activation of AR. In addition, we also tested MageA2 and its MHD domain as specificity controls. We observed that the MHD of MageA6 is sufficient to enhance MageA11-dependent AR activation as MageA6, while MageA2 and its MHD domain failed in regulating AR (**Fig 3D**), suggesting that the MHD domain of MageA6 but not its N-terminal region is required for MageA11 interaction and AR regulation.

### MageA6 expression stabilizes MageA11 protein

**Fig 4:**
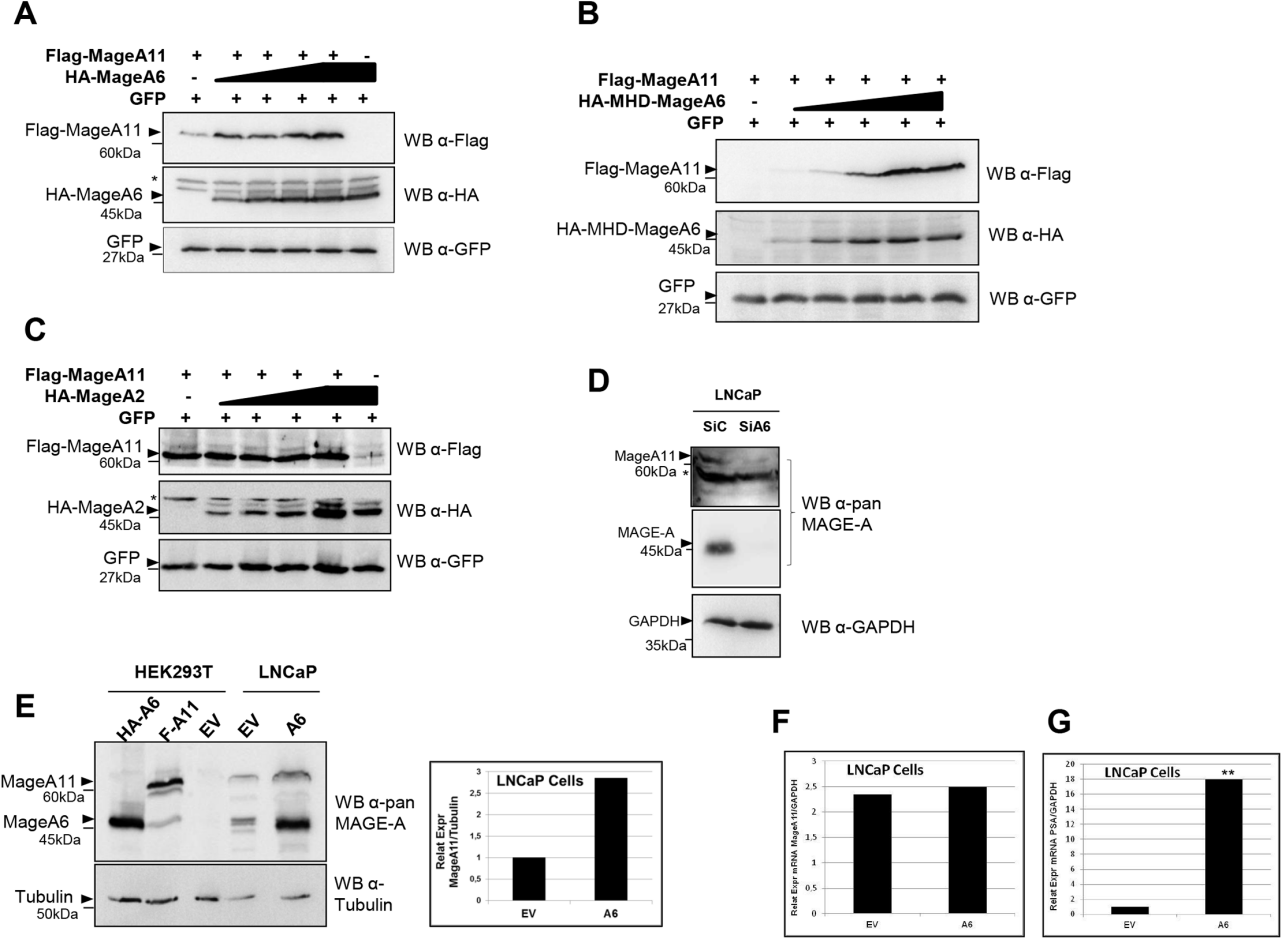
MageA6 expression increases MageA11 protein levels. (A)Western blot showing Flag-MageA11 when co-expressed with increasing quantities of HA-MageA6 (0, 250, 500, 1000 and 1500 ng). GFP expression is the internal control. Membrane was probed with the indicated antibodies. (B) Western blot showing Flag-MageA11 when co-expressed with increasing quantities of HA-MHD-MageA6 (0, 250, 500, 1000 and 1500 ng). GFP expression is the internal control. Membrane was probed with the indicated antibodies. (C) Western blot showing Flag-MageA11 when co-expressed with increasing quantities of HA-MageA2 (0, 250, 500, 1000 and 1500 ng). GFP expression is the internal control. Membrane was probed with the indicated antibodies. (D) Western blot of LNCaP cells silenced (siA6/2) or not (siC) for MageA6 expression. Anti-pan MAGE-A antibody (6C1, Santa Cruz) was used to detect Mage-A proteins. 65KDa band corresponds to MageA11 while 45KDa band could correspond to different Mage-A proteins. GAPDH was used as loading control. (E)Left panel: Western Blot showing the endogenous levels of MageA11 in LNCaP stably expressing MageA6 (A6) or empty vector (EV). Extracts of HEK293T cells transfected with Flag-MageA11 (F-A11), HA-MageA6 (HA-A6) or empty vector (EV) were used as controls. MAGE-A detection was performed with anti-pan MAGE antibody (6C1, Santa Cruz). 65KDa band corresponds to MageA11 while 45KDa band could correspond to different Mage-A proteins. The observed increment in 45KDa band in LNCaP-A6 is caused by MageA6 stable expression. Right panel: quantification of MAGE-A11 vs β-tubulin band intensity corresponding to Fig 4E, lanes 4 and 5. (F) RT-qPCR for the determination of MageA11 mRNA levels in LNCaP-A6 (A6) and LNCaP-EV (EV). MageA11 mRNA was normalized to GAPDH mRNA levels. (G) RT-qPCR for the determination of PSA mRNA levels in LNCaP-A6 (A6) and LNCaP-EV (EV). PSA mRNA was normalized to GAPDH mRNA levels. Error bars indicate mean S.D. Student’s t test was used for statistical analysis. ** p < 0.001. * unspecific band. Triangles show the corresponding protein band and dashes mark the MW.

To understand the basis of MageA6 cooperation with MageA11 in enhancing AR activity, we analyzed the effect of MageA6 expression on MageA11 behavior. No effect on the dynamics of AR translocation to the nucleus was observed upon MageA6 expression (not shown). Instead, we observed that MageA6 expression or its MHD domain correlated to increased MageA11 protein levels (**Fig 4A and 4B**), while MageA2 displayed no appreciable effect **(Fig 4C**). This result suggests that MageA11 stabilization could be linked to enhanced AR activation, since MageA6 but not MageA2 is able to induce AR activity when MageA11 is expressed.

Finally, to verify that MageA6 expression correlates to endogenous MageA11 protein levels, we followed two approaches: MageA6 knock-down by siRNA and MageA6 overexpression in LNCaP cells. To perform the first approach, we transiently transfected LNCaP cells with MageA6 siRNA and endogenous MageA11 protein levels were detected by western blot. MageA6 is an approximately 45 kDa protein, similar to most MAGE-A proteins. MageA11 molecular weight is about 65 kDa, because it displays an extended unusual N-terminal region (see Fig 3D). As shows in **Fig 4D**, endogenous 45 kDa Mage-A proteins were effectively reduced by siRNA and it correlated to diminished MageA11 levels. As stated in Fig 1C, siA6/2 reduces MageA6 and MageA2 expression, but does not affect MageA11 mRNA or protein levels (not shown).

To complete the second approach, we generate LNCaP cells stably expressing MageA6 (LNCaP-A6) or the empty vector (LNCaP-ev) through retroviral transduction. Then, both MageA6 and MageA11 levels were detected in the generated LNCaP cells. As shown in **Fig 4E** (left panel), Western blot analysis detected the expected increase in the 45-kDa MAGE-A protein in LNCaP-A6 cells due to MageA6 overexpression (lane 5) and also an increased 65-kDa MAGE-A protein, corresponding to endogenous MageA11, when compared to LNCaP-ev cells (lane 4). Lysates from HEK293T cells transfected with HA-MageA6 (lane 1) and Flag-MageA11 (lane 2) expression vectors were included as control of molecular weight and antibody reactivity. RT-qPCR determination of MageA11 mRNA levels in LNCaP-V and LNCaP-A6 cells showed no significant changes (**Fig 4F**), indicating that MageA11 increased expression upon MageA6 expression involves protein stabilization rather than mRNA increase. Interestingly, augmented PSA mRNA levels strongly suggest an increased AR activity associated to elevated Mage6 and MageA11 protein expression (**Fig 4G**). All the experiments performed using LNCaP cells in this figure were carried out in the presence of hormone.

**Fig 5:**
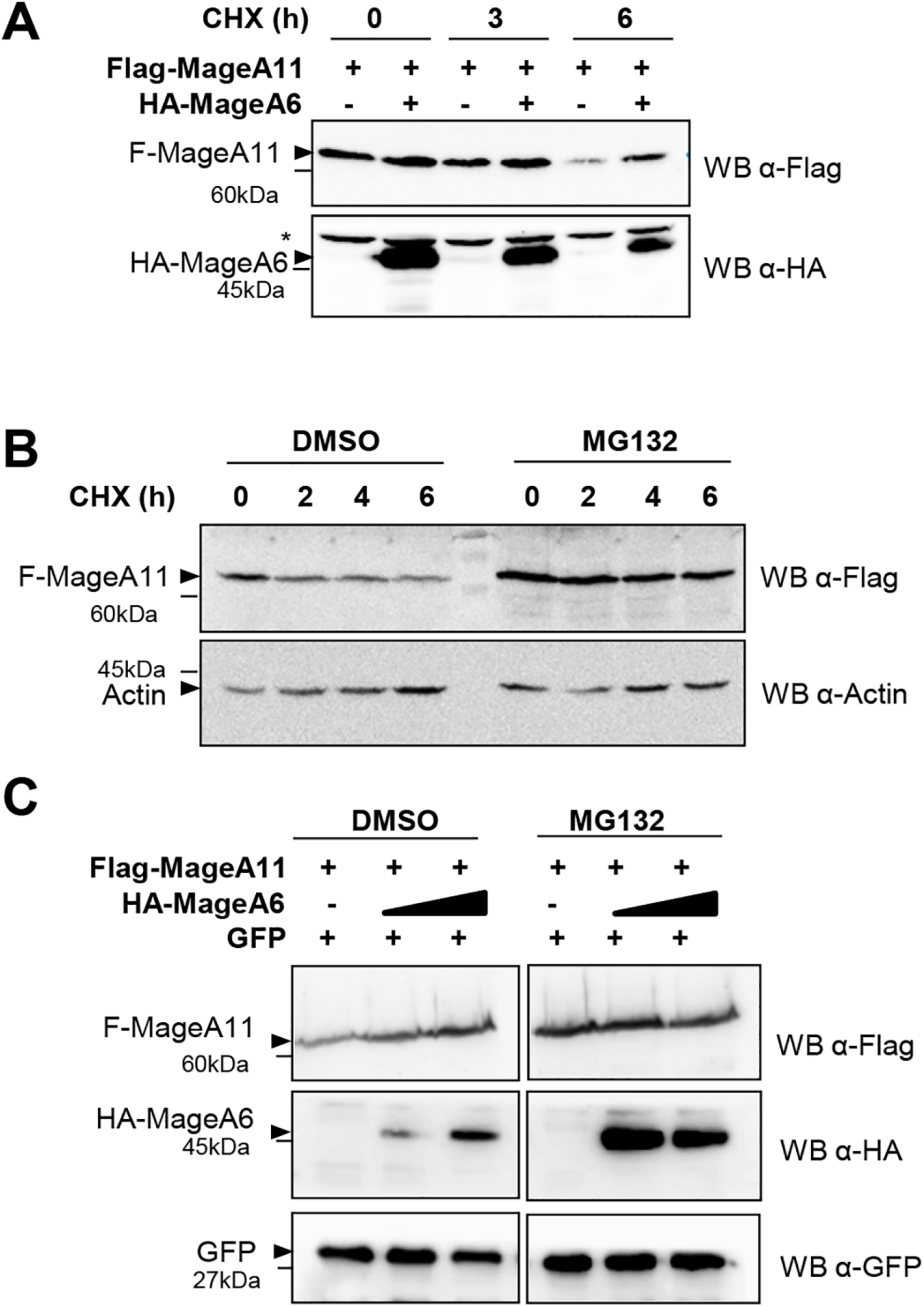
Proteasome-dependent increase of MageA11 by MageA6 expression. (A)Western blot showing Flag-MageA11 alone or when co-expressed with HA-MageA6 after 0, 3 and 6 hours of cycloheximide (CHX) treatment. The membrane was probed with the indicated antibodies. (B) Western blot showing Flag-MageA11 expression in the presence of MG132 (1,5uM for 20h) or absence of MG132 (DMSO) after 0, 2, 4 and 6 hours of cycloheximide (CHX) treatment. The membrane was probed with the indicated antibodies. (C) Western blot showing Flag-MageA11 levels alone or when co-expressed with HA-MageA6 in the presence of MG132 or absence of MG132 (DMSO). GFP expression is the internal control. Membrane was probed with the indicated antibodies. *Unspecific band. Triangles show the corresponding protein band and dashes mark the MW.

We next assessed the effect of MageA6 expression on MageA11 protein levels in cells treated with cycloheximide (translation inhibitor). We observed that MageA6 expression increases MageA11 protein levels independently on protein translation (**Fig 5A**) suggesting that MageA6 decreases MageA11 turnover. Moreover, MageA11 protein stability was also increased in cells treated with the proteasome inhibitor MG132 (**Fig 5B**), corroborating that MageA11 is actively degraded by proteasome. When proteasome activity was inhibited by MG132, MageA6 failed to significantly enhance MageA11 protein levels (**Fig 5C**), demonstrating the relevance of proteasome activity in the mechanism by which MageA6 increases MageA11 protein levels.

### MageA6 expression prevents MageA11 ubiquitinylation

It has been reported that MageA11 is a short-lived protein (t_1/2_ 3,8h approx.) that it is ubiquitinylated in K240 and K245 by a yet unknown E3-ubiquitine ligase, and that such modifications are also important for AR regulation after EGF addition [16]. This fact, prompted us to investigate the effect of MageA6 expression on MageA11 ubiquitinylation.

Noteworthy, recent data has evidenced a close relationship between MAGE and ubiquitinylation. A significant contribution to understand MAGE protein function was the discovery that different MAGE proteins can increase the E3 ubiquitin ligase activity of RING domain containing proteins: MageG1 interacts and activates the E3 ubiquitin ligase NSE, while MageC2 and MageA3/A6 interact and activate the E3 ubiquitin ligase activity of TRIM28/KAP1 protein [15,32]. However, it has been also reported that MageA2 can act as a potent inhibitor the E3 ubiquitin ligase activity of Mdm2 on Mdm4 protein, inducing Mdm4 stabilization [12]. In the same way, it was described that MageC2 can stabilize cyclin E protein by inhibiting the ubiquitinylation activity of the SCF complex [33].

**Fig 6:**
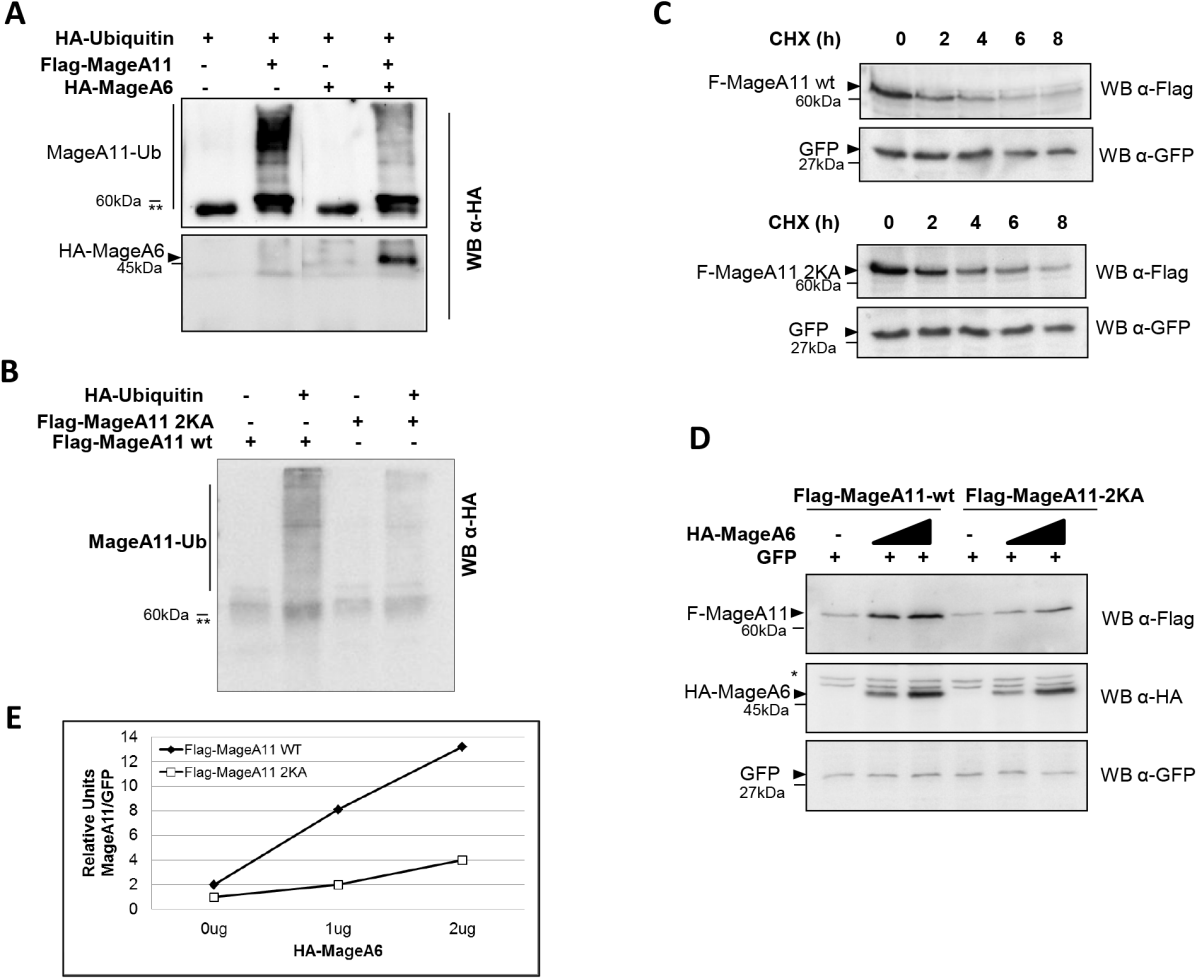
MageA6 interaction with MageA11 prevents MageA11 ubiquitinylation. (A) *In vivo* ubiquitinylation assay in HEK293T cells transfected with Flag-MageA11 and HA-ubiquitin with or without HA-MageA6. Cells were treated with MG132 (10μM) to favor accumulation of ubiquitinylated proteins. Flag-MageA11was immunoprecipitated with anti-Flag antibody (IP anti-Flag). Immunoprecipitated material was assessed by Western blot and probed with the anti-HA antibody. (B) *In vivo* ubiquitinylation assay in HEK293T cells transfected with Flag-MageA11 or Flag-MageA11-2KA (Flag-MageA11^K240A;K245A^) and HA-ubiquitin. Cells were treated with MG132 (10μM) to favor accumulation of ubiquitinylated proteins. Flag-MageA11 was immunoprecipitated with anti-Flag antibody (IP anti-Flag). Immunoprecipitated material was assessed by Western blot and probed with the anti-HA antibody. (C) Western blot showing Flag-MageA11-wt or Flag-MageA11-2KA protein levels of in HEK293T cells transfected with equal amounts of vector DNA as indicated. The day after transfection, cells were incubated with cycloheximide (CHX) for 0, 2, 4, 6 and 8 hours. GFP is an internal control. Membrane was probed with the indicated antibodies. (D) Western blot showing Flag-MageA11-wt or Flag-MageA11-2KA protein levels when co-expressed with increased amounts (0 ug, 1 ug and 2 ug) of HA-MageA6 expressing vector. GFP is an internal control. Membrane was probed with the indicated antibodies. (E) Quantification of Flag-MAGE-A11 or Flag-MageA11-2KA band intensity corresponding to Fig 6D and normalized to GFP. * Unspecific band. ** Immunoglobulin heavy chain. Triangles show the corresponding protein band and dashes mark the MW.

To verify this point we performed an *in vivo* ubiquitinylation assay for MageA11 in cells expressing HA-ubiquitin with or without HA-MageA6. Then, Flag-MageA11 was immunopurified using the anti-Flag antibody and ubiquitinylation was detected by Western blot as a high molecular weight smear reactive to HA antibody. **Fig 6A** (upper panel) shows a lower degree of MageA11 ubiquitinylation when MageA6 is co-expressed, suggesting that it could protect MageA11 from such posttranslational modification that mostly drives proteins for degradation. Accordingly to our results showed in Fig 3A, we also observed that MageA6 was co-purified with MageA11 as it was detected by the anti-HA antibody (**Fig 6A,** lower panel). This result suggests that inhibition of MageA11 ubiquitinylation could be the mechanism by which MageA6 stabilizes MageA11 protein levels.

To verify this hypothesis, we generated a MageA11 expression vector containing mutations on lysine 240 and 245 to alanine (MageA11^K240A;K245A^ also named MageA11-2KA) similarly to that produced by Bai and Wilson [16]. These substitutions should produce an ubiquitinylation defective MageA11 and a more stable protein when compared to the wild type MageA11. We characterized this mutant and verified that MageA11-2KA became less ubiquitinylated under our experimental conditions (**Fig 6B**). Also, mutant MageA11 displays enhanced stability, being clearly detectable 4h after CHX addition, while at this time point the wild-type protein abundance drops significantly (**Fig 6C**). Finally, to understand whether MageA6 could affect the protein levels of the ubiquitinylation defective MageA11, we compared to what extent MageA6 expression could affect MageA11-wt and MageA11-2KA protein levels. Results of co-transfection assays indicated that enhancement of MageA11-2KA protein levels by MageA6 expression was significant lower when compared to the wild-type version (**Fig 6D and 6E**), suggesting that MageA6 could increase MageA11 stability by protecting it from lysine 240 and 245 ubiquitinylation and proteasome-dependent degradation. In agreement to that previously reported by Bai et al, [16], mutant MageA11-2KA did not show effect on AR regulation (not shown).

Since their discovery, MAGE oncoproteins have presented a highly interesting challenge to oncology and cell biology. Due to the conserved amino acid sequence, it was initially hypothesized an overlapped unknown function for these proteins. Ongoing research has demonstrated a degree of complexity, since different MAGE-A proteins can display different functions. In line with this notion, we have recently demonstrated that MAGE-A and MAGE-B proteins also exhibit non-redundant functions [28]. In this study, we have presented evidence for an additional degree of complexity, since two MAGE-A proteins could functionally interact to potentiate a given function, in this case, the hyperactivation of AR, a key factor in prostate cancer. We propose here a novel view on the MAGE networking field, therefore opening the possibilities to the functional collaboration between selected “MAGE-A protein pairs” in a non-redundant way to potentiate a specific oncogenic function. Due to all these novel findings, we stress the relevance to determine the precise identity of MAGE genes expressed in tumor biopsies, since our results suggest that prostate cancers expressing MageA11 or MageA6 and MageA11 could behave differently during anti-hormone treatments.
